# Impact of Mannitol Administration on Postoperative Renal Function After Robot-Assisted Partial Nephrectomy

**DOI:** 10.3390/jcm13216444

**Published:** 2024-10-28

**Authors:** Kazuki Taniguchi, Tomoki Taniguchi, Kentaro Muraoka, Kohei Nishikawa, Yoshinori Ikehata, Kiyoshi Setoguchi, Suguru Oka, Shin Ebara, Akira Fujisaki, Kazuhide Makiyama, Takahiro Inoue, Hiroshi Kitamura, Kazutaka Saito, Shinji Urakami, Tatsuaki Yoneda, Takuya Koie

**Affiliations:** 1 Department of Urology, Daiyukai General Hospital, Ichinomiya 4918551, Japan; glay.apologize@gmail.com; 2 Department of Urology, Gifu University Graduate School of Medicine, Gifu 5011194, Japan; koie.takuya.h2@f.gifu-u.ac.jp; 3 Department of Urology, Yokohama City University, Yokohama 2360004, Japan; kmuraoka@yokohama-cu.ac.jp (K.M.); makiya@yokohama-cu.ac.jp (K.M.); 4 Department of Nephro-Urologic Surgery and Andrology, Mie University Graduate School of Medicine, Tsu 5148507, Japan; kouheini@med.mie-u.ac.jp (K.N.); tinoue28@clin.medic.mie-u.ac.jp (T.I.); 5 Department of Urology, University of Toyama, Toyama 9300194, Japan; ikehata.y0226@gmail.com (Y.I.); hkitamur@med.u-toyama.ac.jp (H.K.); 6 Department of Urology, Dokkyo Medical University Saitama Medical Center, Koshigaya 3438555, Japan; setoguch@dokkyomed.ac.jp (K.S.); kzsaito@dokkyomed.ac.jp (K.S.); 7 Department of Urology, Toranomon Hospital, Tokyo 1058470, Japan; suoka@toranomon.gr.jp (S.O.); shinji.urakami@toranomon.gr.jp (S.U.); 8 Ebara Urology Clinic, Hiroshima 7320053, Japan; shinbone0127@yahoo.co.jp; 9 Department of Urology, Seirei Hamamatsu General Hospital, Hamamatsu 4308558, Japan; akira290z@yahoo.co.jp (A.F.); yonet@sis.seirei.or.jp (T.Y.)

**Keywords:** renal cell carcinoma, robot-assisted partial nephrectomy, mannitol, postoperative renal function

## Abstract

**Background:** This study investigated the effect of mannitol administration on postoperative renal function during robot-assisted partial nephrectomy (RAPN) in patients with renal cell carcinoma (RCC). **Methods:** Patients with RCC who underwent RAPN at eight Japanese facilities between March 2016 and November 2023 were enrolled. In this study, patients were categorized into two groups according to those who received mannitol during RAPN (Group I) and those who did not receive mannitol (Group II). Differences in covariates between the two groups were adjusted using propensity score matching (PSM). **Results:** The study included 1530 patients with RCC who underwent RAPN. PSM was performed on 531 participants in each group. No difference was observed in perioperative outcomes between the two groups in terms of length of hospital stay, surgical outcomes, achievement ratio of Trifecta, and estimated glomerular filtration rate at 28 days, 90 days, and 1 year postoperatively. **Conclusions:** Intraoperative mannitol administration during RAPN for improving renal function may be unnecessary.

## 1. Introduction

Although the total mortality rate of malignant renal neoplasms is increasing in Japan, the age-standardized mortality rates of male patients have been declining since 2016 [1]. This may be due to the increasing number of cancers detected at an early stage as well as advances in surgical treatment, including robotic surgery [1]. For small renal cell carcinomas (RCCs), nephron-sparing surgery, as typified by robot-assisted partial nephrectomy (RAPN), is recommended to preserve renal function [2,3]. Currently, the number of partial nephrectomies is increasing because small RCCs are frequently detected incidentally by screening tests such as imaging studies [4]. Furthermore, the indications for nephron-sparing surgery have been expanding due to advances in surgical techniques such as RAPN [5]. The maintenance of renal function is recognized as an important factor in improving overall survival and oncologic outcomes [6]. Decreased renal function is associated with increased systemic inflammation, anemia, atherosclerosis, left ventricular hypertrophy, increased coagulation activity, increased arterial calcification, and impaired vascular endothelial function [6]. In addition, chronic kidney disease (CKD) has been reported to be an independent risk factor predicting hospitalization, risk of developing cardiovascular events, and mortality [6].

Mannitol is often used during partial nephrectomy to protect renal function, and according to an international survey, mannitol is administered during nephron-sparing surgery in >80% of participating centers [7]. Although mannitol is a water-soluble, naturally occurring sugar alcohol that is rarely absorbed orally, it has free radical-scavenging properties and is thought to increase renal blood flow in administered patients [8,9,10,11]. Furthermore, mannitol administration is thought to be useful for maintaining renal function after renal ischemia, and its renoprotective effects have been evaluated in terms of events related to acute tubular necrosis and ischemia–reperfusion injury [8]. Therefore, the administration of mannitol during renal transplantation has been associated with a lower incidence of acute tubular necrosis and recovery of renal function immediately after kidney transplantation [12]. Conversely, several recent studies reported that mannitol administration during partial nephrectomy does not improve renal function [13,14,15]. A double-blind randomized controlled trial of 65 patients reported that mannitol administration during RAPN did not significantly affect long-term renal function [16]. Mannitol is still customarily administered during RAPN in Japan because there have been few studies on the effects of mannitol administration on the preservation of renal function during RAPN in a Japanese cohort [13].

This retrospective Japanese multicenter cohort study aimed to investigate the effects of mannitol administration on the postoperative renal function during RAPN in patients with RCC.

## 2. Materials and Methods

### 2.1. Patient Population

This retrospective multicenter cohort study was conducted on patients with RCC who underwent RAPN between March 2016 and November 2023 at eight Japanese institutions. Eligible patients were determined to have an RCC < 7 cm in diameter without lymph node metastases or metastases based on clinical staging according to the American Cancer Staging Manual, 8th edition [17]. Patients aged <20 years and those with missing data were excluded from the analysis of this study.

### 2.2. Variables

The following preoperative characteristics of the enrolled patients were collected: age, sex, height, weight, body mass index (BMI), Eastern Cooperative Oncology Group Performance Status (ECOG-PS) [18], clinical tumor stage [19], tumor size and location, and comorbidities such as hypertension and diabetes mellitus. Tumor complexity was evaluated using the R.E.N.A.L. nephrometry score, which comprises (R)adius (tumor size as the largest diameter), (E)xophytic/endophytic properties of the tumor, (N)earness of the deepest portion of the tumor to the collecting system or sinus, (A)nterior/posterior descriptor, and (L)ocation relative to the polar line [19]. The perioperative outcomes of RAPN were assessed in terms of the length of hospital stay (LOS), operative time, estimated blood loss (EBL), warm ischemia time (WIT), and Trifecta achievement rate. Trifecta was determined as negative surgical margins of the removed tumor, WIT < 25 min, and absence of perioperative comorbidities [20]. Mannitol was administered approximately 30 min before renal artery clamping. The dose of mannitol ranged from 20 to 60 g, depending on the patient’s weight or the criteria of each institution. During RAPN, the extent of tumor resection has been determined by means of intraoperative ultrasound [21]. Postoperative renal function after RAPN was estimated based on the proportion of changes in the estimated glomerular filtration rate (eGFR). eGFR was measured using the Modification of Diet in Renal Disease 2 formula (eGFR = 1.94 × serum creatinine level × 1.094 × age × [0.739 for women]) modified for Japanese patients by the Japanese Society of Nephrology [22].

### 2.3. Statistical Analysis

The primary endpoint of the study was whether the intraoperative use of mannitol affected changes in postoperative renal function in patients who underwent RAPN. The secondary endpoint was the association between the intraoperative use of mannitol and other perioperative parameters. The enrolled patients were divided into two groups during RAPN: those who were administered mannitol (Group I) and those who were not (Group II). All statistical analyses were performed using EZR (Saitama Medical Center, Jichi Medical University, Saitama, Japan), which is a graphical user interface for R (The R Foundation for Statistical Computing, Vienna, Austria) [23]. Continuous variables were analyzed using the Mann–Whitney U test, and categorical variables were analyzed using the chi-square test. Propensity scores were calculated using variables that may influence perioperative outcomes (age, sex, BMI, ECOG-PS, clinical tumor stage, tumor diameter, R.E.N.A.L. renal perfusion measurement score, preoperative eGFR, smoking history, hypertension, diabetes, operation time, EBL, WIT) to minimize imbalance between the two groups. Based on the propensity scores obtained, patients in Groups I and II were matched 1:1 using a caliper equal to 0.2 of the standard deviation of the propensity score. Statistical significance for all comparisons was set at a two-sided *p*-value of <0.05.

### 2.4. Institutional Review Board Approval

This study was carried out with the approval of the Mie University Institutional Review Board and the Ethics Committees of the respective institutions (Approval No. H2022-114). As this study was conducted using a retrospective cohort approach, it was assumed that consent was obtained from the patients using an opt-out method instead of obtaining informed consent from each eligible patient. In accordance with Japanese ethics committees and ethical guidelines, written consent is not required for retrospective cohort studies that use existing data that publicly disclose research information. Details of this retrospective study are available, albeit in Japanese only, at https://mie.bvits.com/rinri/publish_document.aspx?ID=3184 (accessed 16 June 2024).

## 3. Results

### 3.1. Patient Characteristics After Propensity Score Matching

A total of 1530 patients were enrolled in the study, of whom 935 received mannitol during RAPN and 595 did not receive mannitol. Table 1 shows the patient characteristics after PSM. In total, 531 patients were assigned to each group. The median and IQR for all patients were as follows: age, 65 years (IQR, 54–73 years); BMI, 24.3 kg/m^2^ (IQR, 22.0–26.9 kg/m^2^); tumor diameter, 25 mm (IQR, 18–33 mm); R.E.N.A.L. nephrometry score, 7 (IQR, 5–8); and preoperative eGFR, 67.8 mL/min/1.73 m^2^ (IQR, 56.9–79.2 mL/min/1.73 m^2^). There were no significant differences in any of the parameters between the two groups.

### 3.2. Surgical Outcomes and Postoperative Renal Function After Propensity Score Matching

The surgical and postoperative outcomes of RAPN after PSM are presented in Table 2. The median and IQR for all patients were as follows: LOS, 7 days (IQR, 6–8 days); operative time, 202 min (IQR, 165.3–243.0 min); and EBL, 11.5 mL (IQR, 0–85.3 mL). Trifecta was achieved in 696 patients (68.8%) after RAPN. The eGFR after RAPN in all patients was 61.8 mL/min/1.73 m^2^ (IQR, 52.1–73.7 mL/min/1.73 m^2^) at 28 days, 61.8 mL/min/1.73 m^2^ (IQR, 51.6–73.0 mL/min/1.73 m^2^) at 90 days, and 60.6 mL/min/1.73 m^2^ (IQR, 51.0–71.9 mL/min/1.73 m^2^) at 1 year after surgery. No significant differences were observed in any of the parameters, including eGFR, between the two groups of patients treated with or without mannitol.

## 4. Discussion

Overall, 434,840 patients were diagnosed with RCC in 2022 worldwide [24]. In the United States, the incidence of RCC increased by approximately 1% annually from 2015 to 2019, whereas the mortality rate of RCC decreased by approximately 2% annually from 2016 to 2020 [24]. The reason for these trends may be the improvement in the prognosis of RCC due to advances in surgical treatments and/or pharmacological therapies [24].

RCC treatment confined to the kidneys include surgical intervention by partial or radical nephrectomy; ablative techniques such as cryoablation, radiofrequency ablation, and radiation; and active surveillance in some patients, especially those with renal masses smaller than 2 cm [24]. Among them, the 5-year cancer-specific survival of partial nephrectomy for renal tumors ≤ 4 cm in diameter, which account for 48% of all the patients, has been reported to be around 94% [24]. Partial nephrectomy for localized renal tumors is not inferior to radical nephrectomy in terms of survival outcomes and preserves renal function significantly better [25]. Therefore, various guidelines recommend open, laparoscopic, or robot-assisted partial nephrectomy for patients with T1 RCC (<7 cm) [2,25,26]. Loss of renal function after partial nephrectomy is influenced by surgical variables such as the amount of parenchymal preservation, ischemia time, medical complications, and preoperative renal function [26]. Globally, 1–2 million people died from CKD in 2017 [27]. Although global all-age mortality from CKD increased by 41–45% between 1990 and 2017, there was no significant change in age-standardized mortality rates [27]. CKD also resulted in 35–38 million disability-adjusted life-years (DALYs) in 2017 with diabetic nephropathy accounting for almost one third of the DALYs [27]. In addition, 1–4 million cardiovascular disease-related deaths and 2.5–3 million cardiovascular disease DALYs have been attributed to CKD [27]. With regard to surgical treatment for patients with RCC, preservation of renal function with partial nephrectomy, which results in an average loss of renal function of approximately 20% after partial nephrectomy, is associated with a lower risk of developing other systemic diseases and lower overall mortality than in patients undergoing radical nephrectomy [28,29]. Therefore, urologists should strive not only to improve oncological outcomes when performing partial nephrectomy but also to preserve renal function as much as possible. In fact, the use of ultrasound guidance during RAPN to determine the extent of renal tumor resection has been suggested to contribute not only to the achievement of negative surgical margins but also to the preservation of normal renal parenchyma [21]. RAPN, currently the mainstay of surgical therapy for small renal tumors, may further expand the indications for surgery without worsening oncological outcomes [25].

To preserve as much renal function as possible during RAPN, the following surgical attempts have been reported: administration of mannitol when clamping the renal artery [7], cooling the kidney before resection of the renal tumor [30], shortening the WIT [31], selective clamping of the renal artery [32], tumor resection in zero ischemia without clamping the renal artery [33,34], and omission of renorrhaphy after renal tumor resection [3,33]. Mannitol administration is considered for the following reasons: to increase plasma volume by increasing renal blood flow, to reduce hypoxic cell swelling secondary to a decrease in hematocrit, and to decrease vascular endothelial cell damage due to postischemic reperfusion by scavenging free radicals [29,35]. Although there are no standardized guidelines for the administration of mannitol to preserve renal function during blood reperfusion after the release of the renal artery clamp and its optimal dosage during partial nephrectomy, owing to the lack of studies with a high level of evidence on the use of mannitol in partial nephrectomy, mannitol administration during partial nephrectomy is commonly used worldwide [7]. Mannitol during renal transplantation is useful for increasing transplant renal blood flow, reducing intravascular cellular swelling and free radical scavenging, and preventing acute tubular necrosis during renal ischemia [10,36]. In addition, mannitol has been reported to be useful in maintaining renal function in RAPN in obese patients with a BMI of ≥30 kg/m^2^ [16]. Therefore, mannitol is now also administered during partial nephrectomy [10,36]. Indeed, mannitol was used in 83% of 92 high-volume centers in a 2012 study on partial and living donor nephrectomy [36]. Mannitol was administered in 78% of partial nephrectomy cases and 64% of living donor kidney harvesting cases for the following reasons: antioxidants in 21%, diuretics in 5%, and both in 74% [36]. The dosage of mannitol varies, with 12.5 g administered in 29.7% of cases and 25 g in 48.6% of cases in partial nephrectomy, which seems to imply that there is no consensus regarding the administration of mannitol [36].

With regard to the timing of mannitol administration, mannitol is more effective when administered at least 15 min before clamping the renal artery [12]. In contrast, the potential risk of developing acute kidney injury (AKI) has also been reported with the use of mannitol [37,38]. The rapid administration of mannitol at 0.25 mg/kg/h causes mannitol-induced AKI within approximately 48 h after administration [38]. The most likely mechanism for the development of AKI is that high doses of mannitol have a vasoconstrictive effect on the renal arteries, which is most likely to cause AKI [38,39]. Therefore, the intraoperative administration of mannitol during RAPN may induce AKI if administered in a similar manner. A multicenter retrospective cohort study examining the association between mannitol administration and subsequent renal function investigated the incidence of de novo stage III CKD (CKD III) and eGFR in patients undergoing RAPN [40]. With a median follow-up of 5 months (IQR, 0.5–19 months), patients who were administered mannitol had a significantly increased incidence of de novo CKD III compared to those who were not (14% vs. 9%, *p* = 0.041) [40]. At the last follow-up time, a significant decrease was also observed in median eGFR in the mannitol-treated group compared with the non-treated group (72.82 vs. 76.06, *p* = 0.039) [40]. In a prospective, randomized, placebo-controlled, double-blind study of 199 patients undergoing partial nephrectomy with preoperative eGFR > 45 mL/min/1.73 m^2^ from 2012 to 2015, participants were randomized to receive either mannitol (12.5 g) or placebo intravenously within 30 min before renal artery clamping [9]. The results showed no significant clinical benefit of mannitol administration during partial nephrectomy compared with the placebo group and concluded that renal function could be maintained with adequate replacement fluid administration without mannitol during partial nephrectomy [9]. In a subsequent post hoc analysis, it was also evaluated whether mannitol administration was associated with long-term postoperative eGFR [41]. There was no significant benefit with or without mannitol administration on eGFR at either 6 months or 3 years after partial nephrectomy [41]. In a Japanese retrospective cohort study of patients with a single kidney undergoing partial nephrectomy, the postoperative eGFR, its rate of decrease, and the incidence of AKI requiring dialysis with or without intraoperative mannitol administration were compared [15]. The administration of mannitol did not significantly affect eGFR or its rate of decrease at any time point within 6 months after surgery, nor did it affect the incidence of AKI requiring dialysis [15]. These findings suggest that there may be no benefit in preserving renal function with the administration of mannitol in partial nephrectomy [15]. Currently, few studies have examined the effect of mannitol administration during RAPN on postoperative renal function in Japanese patients. However, the results of this study showed that mannitol administration during RAPN did not improve renal function, even in Japanese patients.

Several limitations exist in this study. First, this was a retrospective study using data from multiple centers, resulting in potential bias due to differences in the surgical techniques at each center, including the approach to RAPN, timing and method of clamping the renal arteries, location of the safety margin around the renal tumor, and suturing of the renal parenchyma after tumor removal. Therefore, differences in surgical procedures at each institution may have the potential for bias. Second, because of the relatively short follow-up period, long-term changes in renal function were unknown. Third, the number of cases experienced by surgeons who performed RAPN and the difficulty level of the surgery, depending on the location of the tumor, were different. Therefore, the presence or absence of mannitol administration alone did not seem to have an effect on postoperative renal function. Fourth, most of the articles cited in this study were related to open partial nephrectomy. The reason for selecting these papers is that there are currently few reports investigating the effect of mannitol on renal function in RAPN. Finally, caution must be exercised when interpreting the results of this study because the mannitol dosage, timing of administration, and hourly dosage varied among institutions and surgeons.

## 5. Conclusions

To our knowledge, this is the first Japanese cohort study to evaluate the association between postoperative renal function and the presence or absence of mannitol administration during RAPN. In this study, using PSM, mannitol administration during RAPN did not contribute to an improvement in renal function. Therefore, intraoperative mannitol administration during RAPN may be unnecessary to improve renal function.

## Figures and Tables

**Table 1:** Comparison of preoperative covariates after propensity score matching in the two groups divided by mannitol use during robot-assisted partial nephrectomy.

	Group I	Group II	*p*
Number of patients	531	531	
Age (year, median, IQR)	66.0 (54.0–73.0)	63.4 (54.7–72.0)	0.184
Sex (number, %)			0.597
Male	369 (69.5)	360 (67.8)	
Female	162 (30.5)	171 (32.2)	
Body mass index (median, IQR)	24.3 (22.1–27.0)	24.3 (21.9–26.8)	0.574
ECOG-PS (number, median, IQR)	0 (0–0)	0 (0–0)	0.098
Clinical Tumor stage			0.852
T1a	467 (87.9)	464 (87.4)	
T1b	64 (12.1)	67 (12.6)	
Tumor diameter (mm, median, IQR)	25.0 (18.0–34.5)	25.0 (18.0–32.0)	0.466
R.E.N.A.L nephrometry score (number, median, IQR)	6.0 (5.0–8.0)	7.0 (5.0–8.0)	0.532
Preoperative eGFR (mL/min/1.73 m^2^)	67.2 (58.0–77.3)	68.4 (56.0–80.5)	0.443
Smoking history (number, %)	257 (48.2)	252 (47.5)	0.806
Hypertension (number, %)	262 (49.3)	252 (47.5)	0.581
Diabetes mellitus (number, %)	122 (23.0)	109 (20.5)	0.372

Group I, patients who received mannitol during surgery; Group II, patients who did not receive mannitol during surgery; IQR, interquartile range; ECOG-PS, Eastern Cooperative Oncology Group Performance Status; eGFR, estimated glomerular filtration rate.

**Table 2:** Comparison of surgical outcomes and postoperative renal function by propensity score matching after robotic-assisted partial nephrectomy with and without mannitol administration.

	Group I	Group II	*p*
Number of patients	531	531	
Length of hospital stay (days, median, IQR)	7.0 (6.0–8.0)	7.0 (7.0–8.0)	0.640
Operation time (min, median, IQR)	202.0 (169.0–243.5)	202.0 (155.5–242.5)	0.123
Estimated blood loss (mL, median, IQR)	10.0 (0–100.0)	15.0 (3.0–62.0)	0.114
Warm ischemia time (min, median, IQR)	19.0 (14.0–23.0)	19.0 (14.0–24.0)	0.499
Achievement rate of Trifecta (number, %)	332 (67.1)	364 (70.4)	0.282
eGFR 28 days after operation (mL/min/1.73 m^2^, median, IQR)	62.6 (53.7–72.0)	60.9 (49.7–75.6)	0.306
eGFR 90 days after operation (mL/min/1.73 m^2^, median, IQR)	61.7 (53.0–69.7)	62.1 (50.1–74.5)	0.644
eGFR one year after operation (mL/min/1.73 m^2^, median, IQR)	60.0 (51.2–71.1)	61.9 (50.1–74.1)	0.353

Group I, patients who received mannitol during surgery; Group II, patients who did not receive mannitol during surgery; IQR, interquartile range; eGFR, estimated glomerular filtration rate.

## Data Availability

The data presented in this study are available on request from the corresponding author. The data are not publicly available due to privacy and ethical reasons.
